# Influenza Virus Neuraminidase: Structure and Function

**Published:** 2009-07

**Authors:** Y.A. Shtyrya, L.V. Mochalova, N.V. Bovin

**Affiliations:** 1 Shemyakin and Ovchinnikov Institute of Bioorganic Chemistry, RAS

## Abstract

The structure of the influenza virus neuraminidases, the spatial organization of their active site, the mechanism of carbohydrate chains desialylation by neuraminidase, and its role in the influenza virus function at different stages of the viral infectious cycle are considered in this review. Data on the neuraminidase substrate specificity and different approaches in studying the activity of this enzyme are summarized. In addition, data on neuraminidase inhibitors (as antivirals) are provided, along with considerations on the mechanisms of resistance of modern influenza viruses to those antivirals.

The influenza virus is an enveloped (-)RNA containing a virus with a segmented genome, and its genetic material is coded by eight RNA-segments. All RNA segments are packed in a nucleocapsid protein, and a complex of polymerase proteins is attached to each of the genomic segments. Those RNA-protein complexes are packed in a lipoprotein envelope lined from the inside with a matrix protein, with haemagglutinin, neuraminidase, and M2 proteins exposed on the outer surface of the viral particle.

Neuraminidase is an exosialidase (EC 3.2.1.18) which cleaves α-ketosidic linkage between the sialic (N-acetylneuraminic) acid and an adjacent sugar residue [1]. The amino acid sequence of NA is coded by the 6th RNA segment. Nine subtypes of NA are described for influenza A, whereas only one NA subtype was revealed for the influenza viruses B and C [2]. Nine subtypes of influenza A NA are divided into two phylogenic groups. The first group consists of the neuraminidases of N1, N4, N5 and N8 subtypes, and the second one consists of N2, N3, N6 N7 and N9 subtypes [3].

The enzyme of the influenza C virus does not belong to the neuraminidase group. It promotes the O-deacetylation of the N-acetyl-9-O-acetylneuraminic acid, i.e. it belongs to the esterase family and will not be considered in this review.

The influenza virus NA executes several functions. Firstly, its activity is required at the time of the budding of newly formed viral particles from the surface of the infected cell, to prevent aggregation of viral particles. In addition, NA cleaves neuraminic acid residues from the respiratory tract mucins; by doing so, it facilitates viral movement to the target cell. Those functions will be considered further in more detail.

## NEURAMINIDASE STURCTURE

### 

The polypeptide chain of the influenza virus NA comprises 470 amino acid residues. The three-dimensional structure of NA consists of several domains: the cytoplasmic, transmembrane, "head," and also "stem," connecting the head to the transmembrane domain.

On the virion surface, NA resembles a homotetramer of a mushroom shape: head of 80*80*40 Å on the thin stem, 15 Å wide and from 60 to 100 Å long [2]. The molecular mass of the monomer is ≈ 60 kDa, and ≈ 240 kDa for the tetramer [1]. One viral particle has approximately 50 tetramers. Tetramers can form clusters on the viral surface [4]. The three-dimensional structure has been revealed for N1, N2, N4, N8, N9 and В NA [1, 3, 5, 6, 7]. Notwithstanding that NA types A and B homology cover only 30 %, their three-dimensional structures are virtually identical [6].

### Head

The enzyme active site and calcium binding domain, which stabilizes the enzyme structure at low pH values, are situated in the head of NA [2; 8].

**Fig. 1.**
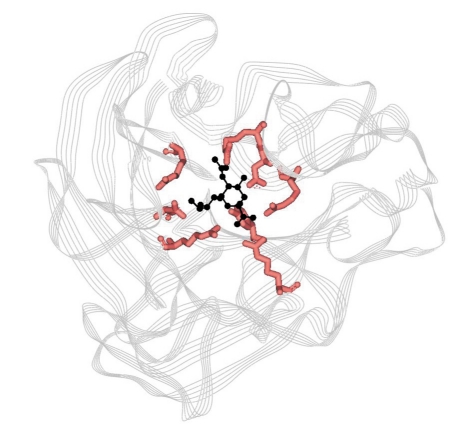
Active site of influenza virus A neuraminidase (N2 subtype) in complex with Neu5Ac2en (2-deoxy-2,3-didehydro-N-acetylneuraminic acid). Neu5Ac2en is presented in black, functional a.a. of the active site – red

Homology between the strains inside one subtype attains about 90%, whereas homology between subtypes is 50%, and 30% between А and В types [9]. A.a. region 74-390 is the most conservative (N2 numbering)1. Residues, which account for the catalytic function of the enzyme (Arg118, Asp151, Arg152, Arg224, Glu276, Arg292, Arg371 and Tyr406, Figure 1), are constant for all NA subtypes of influenza A and also for influenza B NA. This works also for amino acids, which form the dimensional structure of the active site: Glu119, Arg156, Trp178, Ser179, Asp198, Ile222, Glu227, Glu277, Asp293, and Glu425. Asparagine residues, which form the glycosylation site, are strictly conservative (specifically, Asn146), proline and cysteine residues, which provide the required folding of the polypeptide chain and stabilize the 3-dimentional structure of the molecule, are also quite conservative [2].

1 As amino acid sequences of different neuraminidases differ from one another by insertions and deletions, it is common practice to highlight NA subtype according to which the numbering of amino acids is done, usually, as in this case N2 subtype numbering is used.

The calcium binding site, which is located inside the molecule (particularly under the active site, if it is placed in accordance with the picture provided) is formed by the oxygen of the main chain residues 297, 345 and 348, as well as by the oxygen of the side chain of Asp324 [1, 6]. Additionally, this site is formed by a.a. 293, 347, 111-115 and 139-143 [8].

The second neuraminic acid binding site, the so called HBsite, was found in N9 neuraminidases [10]. The a.a. sequence of this site is highly conservative among avian influenza viruses. This site is formed by three NA loops:

367 − 372, which is involved in neuraminic acid binding via serine residues 367, 370 and 372;

400 − 403, which interacts with the substrate via the side chain of asparagine 400, the carbonyl oxygen of the main chain of asparagines, and tryptophan 403;

430 – 433, which interacts with neuraminic acid via the ε-amino group of lysine 432.

All six above-mentioned conservative amino acids were found only in N9 NA. Avian influenza virus NAs of other subtypes usually lack lysine 432, but its absence does not interfere with their haemoadsorption activity. Human influenza virus neuraminidases usually lack the HB-site a.a. sequence. At the same time, two early isolates of human influenza viruses of the Н2N2 subtype (RI+/57 and A/Leningrad/ 134/57) have the HB-site "frame" (serine triplet and tryptophan) [10, 11], which might be indicative of elimination of this site in the course of the influenza virus adaptation to replication in humans.

The function of the HB-site has yet to be clarified. It has been suggested that it may play the role of an alternative neuraminic acid binding site, in other words, function as a surrogate of the influenza virus HA; this assumption is based on the existence of viruses with combined HA and NA functions in one protein molecule, such as the ND virus. The HB-site, described earlier, is common for the NAs of viruses which HA interacts with α2-3-sialylated carbohydrate chains (i.e. avian and equine influenza viruses), at the same time, the key amino acid positions of this site are changed in viruses with α2-6-specificity (human, swine, and poultry H9N2 viruses). It is worth mentioning that the H9N2 influenza viruses isolated from poultry in Hong Kong and viruses of H2N2 and H3N2 subtypes, which caused human pandemics, have similar changes in the HB-site sequence. This data allows one to suggest that some species of poultry may act as intermediate hosts in the influenza virus transfer from its natural reservoir (waterfowl population) to humans [11].

### Three-dimensional structure 

The three-dimensional structure of cytoplasmic, transmembrane and stem domains has not been determined yet (due to the features of the enzymes, which are used for cleavage of this membrane protein from the virion, the crystallized region starts at residues ~74-77) [6]. There is speculation about the presence of an α-helix motif in the uncrystallized structure, which has been supported by cryoelectron microscopy [4]. Therefore, one can unequivocally judge only the chain folding of the head region of the enzyme (as part of the tetrameric structure). The NA's head region consists of one big domain, which is formed by six identical antiparallel β-sheets (motifs) organized in the form of a propeller-like structure. Loops connecting the motifs and loops between every second and third strain of each motif are of ultimate importance for the enzyme [2]. Loops are the most variable parts of the structure of all NAs; they vary in length and can even have some arranged elements typical of the secondary structure. For example, loops of N9 NA have some α-helix regions: residues 106 – 110 form one spiral turn (α), located above the С-terminus of the polypeptide chain, which consecutively forms another α-helix turn, and a.a. 144 – 146 of the neighboring subunit forms the (310) helix. The 310 helix and two chains (106 − 110 and C-terminus) form the antiparallel layer [6]. The loop connecting the fourth and the fifth motifs is the longest one and is stabilized by a disulfide bridge located between Cys318 and Cys337; it also has the conservative ion pair Asp330-Arg364 and the Ca^2+^ binding site [1].

### Glycosylation

Carbohydrate chains are attached to Asn residues located in the different regions of the NA's head. In particular, glycans attached to asparagines 86 and 234 are headed towards the lipid membrane close to the stem; those attached to Asn146 – are headed from the membrane and are located in close proximity to the active site; finally, glycosylation Asn200 is located on the side surface in the region of the subunits contact. Short oligomannose type chains were found at residues Asn86 and Asn200. Carbohydrate chains of a complex type are attached to Asn146 and Asn234. The glycosylation site at Asn146 is conservative for all NAs, and this carbohydrate chain differs from all other carbohydrates found in all influenza virus glycoproteins: it carries the О-4 sulfated N-acetylgalactosamine [1]. Asn146 glycosylation seems to have a regulatory function, because it is known that the lack of this glycosylation site determines the influenza virus А/WSN/33 (Н1N1) neurovirulence. It has been shown that the carbohydrate chain at Asn146 affects NA enzymatic activity, causing a 20-fold decrease in activity [12]. Deletion of the glycosylation site at a.a. 144 of N8 NA (А/duck/ Ukraine/1/63) causes changes in its substrate specificity profile NA [13], and absence of glycosylation sites at a.a. 83 and 398 causes incorrect molecule folding.

### Disulfide bonds

There are eight conservative disulfide bonds in the NA structure, and one additional bond in the N2, N8, and N9 subtypes. The invariance of disulfide bonds confirms their importance in the formation of a stable NA structure. It is assumed that, because of its proximity to the symmetry axis of the tetramer, the uncoupled Cys161 of N1 NA takes part in the coupling of subunits. The tetramer assembly mechanism is not universal: for instance, in influenza virus B neuraminidases disulfide bonds are formed by Cys54, whereas Cys78 takes part in polypeptide chains binding (N2 numbering) [2].

### Active site structure

The Neu5Ac binding site is located above the first strands of the third and the fourth motifs in a big loop on the NA surface. The active site is located at the N-terminal end of central parallel strands [2]; (Fig.1). It is a cavity 16Å in diameter and 8 to 10Å in depth, located 32Å from the tetragonal axis. This site is surrounded by twelve flexible loops, which go upwards from that axis [6].

The enzyme active site consists of functional amino acid residues Arg118, Asp151, Arg152, Arg224, Glu276, Arg292, Arg371, and Tyr406, and structural amino acid residues Glu119, Arg156, Trp178, Ser179, Asp (or Asn in N7 and N9) 198, Ile222, Glu227, Glu277, Asp293, and Glu425.

Functional a.a. are in direct contact with sialic acid, the product of the enzymatic reaction, and they all form polar contacts with it, excluding Arg224, whose aliphatic part forms a nonpolar contact with the glycerol fragment of the Neu5Ac residue [9] (Fig.1).

Recent X-ray studies of neuraminidases from the first phylogenic group have shown that, in comparison with neuraminidases from the second phylogenic group, they have a slightly different structure of the polypeptide chain around the enzyme active centre. In particular, there is a cavity in close proximity to the active site, which is formed by a change in the dimensional orientation of "loop 150." These structural differences allow the launch of the development of influenza virus NA inhibitors, which would specifically interact only with NAs of the first phylogenic group, in particular with the NA of influenza viruses of the H5N1 subtype [3].

## REACTION MECHANISM

**Scheme 1:**
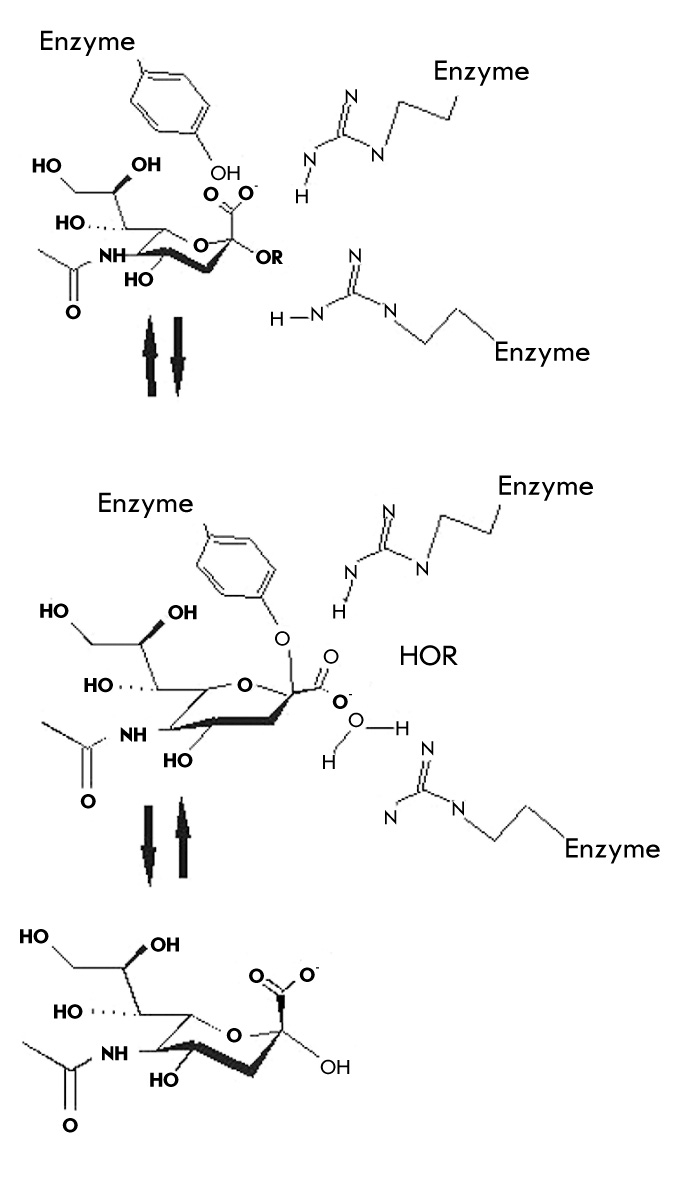
Mechanism of substrate desialylation by influenza virus neuraminidase (according to [7], [15], and [16].)

The NA's reaction mechanism (Scheme 1) was proposed based on the results of structural studies of the crystallized protein [7].

Formation of the oxocarbonium ion at the С2 atom of Neu5Ac is the key step in the hydrolysis of the oligosaccharide substrate. After the introduction of the Neu5Ac residue into the active centre, Neu5Ac conformation changes from chair to half-chair, i.e. the oxocarbonium ion is formed, due to strong ionic interactions between the carboxylate of the substrate and the guanidine groups of the arginines 118, 292 and 371, eventually leading to glycosidic bond cleavage. The molecule of aglycone leaves the enzyme active site with glycosidic oxygen, protonated by the solvent. Multiple contacts between the intermediate product and the a.a. of the active site (Tyr406 and Asp151 are of minor importance) stabilize the positively charged oxocarbonium ion with preservation of the planar carbon at С2. Neu5Ac2en, in which the C2 atom is in sp2-form, mimics the intermediate reaction product in planar conformation [6]. At this stage of the reaction, the neuraminic acid residue is covalently bound to the hydroxyl group of Tyr406, which is characteristic of all exosialidases [15, 17]. Hydroxylation of the oxocarbonium ion with the solvent and product leaving the enzyme active site in the form of Neu5Ac are the limiting stages of the catalytic reaction. It is worth mentioning that there are no significant changes in the coordinates of the NA active site during the reaction [18].

The presence of invariant residues in the active site, the similarity of the structural organization, and the architecture of complexes with Neu5Ac and with Neu5Ac2en allow to assume that the mechanism of NA functioning for the A and B influenza viruses is identical [6].

## NEURAMINIDASE INHIBITORS AND MECHANISM OF ANI-DRUG RESISTANCE

**Fig. 2.**
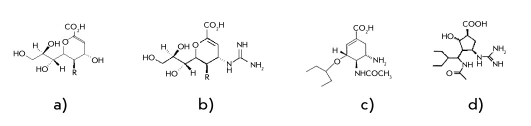
Structure of а) Neu5Ac2en, b) Zanamivir, c) Oseltamivir, d) BCX-1812 (preamivir), where R=NHAc

The structure of the neuraminidase active site is strictly conservative not only between subtypes, but also between the types of the enzyme, which points to the importance of all its components and the evolutionary stabilized functioning of this system. This observation has allowed to design an NA inhibitor for the influenza virus which mimics the transition state of the hydrolysis reaction, and Neu5Ac2en (Fig.2а), 4-guanidino-Neu5Ac2en, which is now widely used under the trade name zanamivir [14] (Fig. 2b).

The success of this drug has initiated a number of studies aimed at designing new NA inhibitors. The main structural elements of the new class of inhibitors (without the oxygen atom in the cycle) are cyclohexane and cyclopentane. One of those structures is the (3S,4R,5R)-3-amino-4-acetamido-5-(1-ethylpropoxy)-1-cyclohexene-1-carboxylic acid (oseltamivir or Tamiflu) (Fig. 2c). The structure of this molecule is adjusted to coordinates of the amino acids, which interact with the glycerol chain of Neu5Ac2en [20]. Successful use of this drug has stimulated the development of new NA inhibitors with hydrophobic groups [21].

Besides, a NA inhibitor on the base of a cyclopentane structure has been developed; it has all the functionally important parts of zanamivir (carboxyl, acetamide, C4-hydroxyl) which fit into the NA active centre. BCX-1812 (preamivir) (Fig. 2d) retains its activity towards zanamivir-resistant influenza viruses [22, 23]. At present, preamivir analogs are at the development stage.

Zanamivir and oseltamivir are already used as drug products, whereas BCX-1812 has entered the last phase of clinical trials.

Until recently, it was considered that active uncontrolled use of zanamivir and oseltamivir would not have a significant influence on the development of resistance in influenza virus strains. That is, even if resistant strains emerge, they would not be able to replicate in the absence of the inhibitor [24]. The number of resistant viruses isolated in clinical trials accounted for less than 1%, same as their presence among seasonal influenza virus isolates worldwide.

However, in January 2008 this situation changed dramatically: some H1N1 influenza viruses developed resistance to oseltamivir due to a mutation His274Tyr in NA [25], and in the epidemic season of 2008-2009 resistance was up to 100% among viral isolates (according to http://ecdc.europa.eu); it is typical that those isolates preserved sensitivity to zanamivir. The mutation His274Tyr had been spotted in studies of resistance in vitro and in vivo, as well as in clinical isolates [26]. Nevertheless, it is still too early to stop usage of this drug, because according to the most recent data, the prepandemic influenza virus of the H1N1 subtype (A/California/11/2009), which is encountered at the moment in humans, is still susceptible to oseltamivir (according to data from the Center for Disease Control and Prevention, USA (www.cdc.gov). This leaves us with hope that the strain of the influenza virus that will cause the next pandemic might be susceptible to this NA inhibitor.

## NA FUNCTIONAL ACTIVITY

### 

There is data indicating that NA is relevant at different stages of infection. Firstly, it is considered that it helps the virus approach the target cells by cleavage of sialic acids from respiratory tract mucins [26]. Secondly, it may take part in the fusion of viral and cell membranes [27]. Thirdly, it facilitates budding of new virions by preventing their aggregation, caused by the interaction of the HA of the first virus with the sialylated glycans of the second one [27]. In addition, there is data suggesting that NA amplifies HA haemagglutinating activity by cleavage of the terminal neuraminic acid residues of the oligosaccharides surrounding the receptor-binding site of HA [28].

One of the most interesting features of the influenza virus is the coexistence of two proteins whose functions are to some extent contradictory, namely: haemagglutinin, which has a receptor-binding function; and neuraminidase, which has a receptor destroying function. Since both of these proteins recognize terminal neuraminic acid residue, this brings up the question of their cooperation or, on the contrary, their competition for receptor/substrate, and of the role of their relations in viral life cycle. Studies of the viruses resistant to NA inhibitors, artificial viral reassortants (which have HA and NA of different origins), and virus particles designed by means of reverse genetics, which lack NA or HA activity, show that the NA and HA of the influenza virus act in concert and their evolution proceeds interdependently [29-35]. Also, it raises a question as to their oligosaccharide specificity, because Neu5Ac-terminated oligosaccharide chains in viral hosts are quite diverse.

### Methods for determination of NA activity

**Fig. 3.**
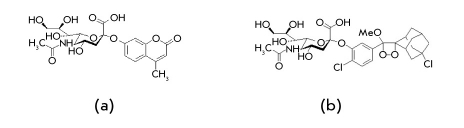
Structure of MU-Neu5Ac (а) and NA-Star (b)

One of the most popular substrates for NA activity determination is MU-Neu5Ac or MU-NANA (Fig. 3a). The method based on the use of this substrate was proposed [36] first as an alternative to colorimetric or radioactive methods. After cleavage of the neuraminic acid, MU-Neu5Ac forms a fluorophore which is activated by light at a wavelength of 360 nm, and its fluorescence maximum is achieved at pH 10. The closest analog of MU-Neu5Ac is the 4-trifluoromethylumbelliferyl- α-D-N-acetylneuraminic acid, whose fluorescence maximum is located in the neutral pH range. High fluorescence intensity (10-fold higher than for MUNeu5Ac) is useful in studies of low-activity neuraminidases [37].

The sensitivity of the chemiluminescent method of NA activity determination [38] is by a factor of 100 higher than that of MU-Neu5Ac-assay, and «NА-Star» is used as a substrate (Fig. 3b). The main disadvantage of this method is the short lifetime of the product of chemiluminescent hydrolysis, which has to be recorded within 5 minutes.

The other group of methods is based on cleavage of the neuraminic acid from high molecular weight substrates, such as fetuin, the α1-acid glycoprotein or whole erythrocytes. The amount of free neuraminic acid is usually determined after cleavage [39]; the most convenient procedure for assay of Neu5Ac allows for conducting measurements in the presence of the sialylated substrate [40].

An alternative procedure is based on assay of the second product of the hydrolysis, the desialylated glycoprotein, with the help of lectin (for example, Peanut agglutinin), which is specific for the unmasked terminal galactose [41, 42]. Carrying on with this analytical procedure requires great accuracy in control preparation, as every glycoprotein originally has terminal β-Gal residues.

### Methods for determination of NA substrate specificity

The substrate specificity of NA is its ability to discriminate between sialic acids (for example, Neu5Ac and Neu5Gc) and linkage type with the next residue (2-3, 2-6 or 2-8), as well as the ability to identify internal regions of the oligosaccharide chain. In particular, the following structures have been used for the determination of NA substrate specificity:

• free trisaccharides (3' SiaLac or 6'SiaLac) [43 - 45],

• glycoproteins containing only 2-3, or only 2-6-linked neuraminic acid [45, 46],

• glycoproteins or erythrocytes oversialylated with the aid of 2-3- or 2-6- sialyltransferases [47].

Methods based on the use of those substrates achieve only one of the listed goals; in particular, they allow to study specificity at the level of Sia2-3Gal or Sia2-6Gal. More broad specificity can be studied with the use of an analytical procedure which employs a number of synthetic substrates. In [42], a panel of three oligosaccharides was used: 3'SiaLac, 6'SiaLac and 6'SiaLacNAc, in the form of polyacrylamide conjugates; and neuraminidase activity was measured by lectin, specific for galactose residues, which appear as the result of NA action (see above). A new simple and sensitive method for NA specificity determination has been developed recently [48]. It is based on the use of BODIPY-labeled sialyloligosaccarides. The fluorescent label is covalently bound to the oligosaccharide (3'SiaLac, 3'SiaLacNAc, SiaLec, SiaLea, SiaLex, 6'SiaLac, 6'SiaLacNAc) via spacer, i.e. it is at some distance from the cleavage site. Stability, relative hydrophility, electroneutrality, small size, and ability to use standard fluorescent filter for detection are the advantages of this label. The method is based on a quantitative separation of the electroneutral product of the reaction and the negatively charged substrate, separation is performed either on a microcartrige with an anion-exchange sorbent or microplates, the semipermeable bottom of which consists of an anion-exchange material. For greater reliability one may quantify the amount of the reaction substrate, along with the quantity of the reaction product. The high sensitivity of the method makes it possible to work with low substrate concentrations (10-11 mol), as well as with low virus concentrations. High accuracy (more than 95%) and good reproducibility (98%) of the new method allow to study the kinetics of enzyme-substrate interactions. Studies of desialylation kinetics, in particular the reaction velocity and its dependence on substrate and enzyme concentration, is important for understanding the reaction mechanism, as well as for the choice of the correct concentration range. In turn, the correct range allows to study desialylation specificity in cases when the NA quantity in the test sample is unknown [49]. It is worth mentioning that only this approach allows to study many aspects of NA substrate specificity (see above), namely to study the influence of the sialic acid type, the type of linkage between the sialic acid and the next sugar, and the influence of the inner glycan sugars.

### Functional features of some influenza virus neuraminidases

As already mentioned above, high molecular weight substrates, along with low molecular weight substrates, can be used for studying NA activity and specificity. Low molecular weight substrates allow to study the reaction mechanism and desialylation kinetics without the complications of multivalent interactions (NA is a tetramer) and the possible influence of HA, which interacts with multivalent conjugate 3 – 5 orders of magnitude better than with the monomeric one [50]. High molecular weight substrates appear to be a more accurate model for studying natural interactions; that is when there is a necessity to account the NA tetrameric organization, the clustering of NA molecules on the cell surface, and the involvement of the second surface glycoprotein, HA, which is present on the viral surface in larger amount.

Investigation of the evolution of the influenza virus NA substrate specificity for viruses isolated from humans, and its comparison with the substrate specificity of influenza virus NAs isolated from different hosts, such as ducks and pigs, is of great importance. The first undertaking could shed light on the question of the unique character of pandemic strains, while the second could help detect in advance the properties of the enzyme which facilitate the crossing of the interspecies barrier.

The specificity of N2 subtype NA of human influenza viruses has gradually changed from 3'SiaLac (H2N2 strains isolated in 1957) to dual specificity, 3'SiaLac/6'SiaLac (strains from 1972 to 1987). Hydrolytic activity towards 6'SiaLac was identified only for viruses isolated in 1967 and further, and starting from 1972 isolates an increase of activity towards this substrate was registered [46].

It has been shown recently that N2 influenza viruses are highly active towards 3'SiaLac, while their activity towards 6'SiaLac varies from extremely low (avian and early human isolates) to high (swine and latter human isolates). It has been shown that NA activity towards 6'SiaLac depends also on the host type and, for human viruses, on the year of isolation [45].

For N1 strains isolated in the 70-80s [43, 44], it was shown that their neuraminidase equally recognizes 3'SiaLac and 6'SiaLac.

Data on the substrate specificity of N1 and the N2 NAs of several duck, swine and human influenza virus isolates were obtained with the use of BODIPY-labeled synthetic oligosaccharides [48-51]. All of the studied NAs desialylated α2-3-substrates better then α2-6 ones. In the case of viruses with N1 neuraminidase, α2-3/α2-6 activity factor was ~60 for duck viruses, ~20 for swine viruses, and ~4 for human viruses. In case of H9N2 influenza viruses, similar α2-3/α2-6 relations were found for the duck virus, whereas this relation for viruses isolated from poultry is in a range from 30 to 15, and for swine virus ~6, finally, for human isolate ~10. For all the studied NA, it has been shown that they discriminate the fine structure of α2-3-substrates, that is they discriminate between the structures of the inner parts of oligosaccharides.

With the use of the polyacrylamide conjugates of 3'SiaLac, 6'SiaLac and 6'SiaLacNAc, it has been shown that most of the viruses (H1N1 and H3N2 subtypes) propagated on embryonated chicken eggs and MDCK cells, preferably hydrolyze 3'SiaLac, whereas VER O-isolates of the same viruses preferably hydrolyze 6'SiaLacNAc. In summary, the nature of the host cell line used for virus accumulation influences NA substrate specificity [42]. The reason for this effect remains unknown.

It is difficult to compare the results of substrate specificity studies conducted by different authors due to the use of both different influenza virus strains and different substrates in varying concentrations. For example, the Kobasa [45] group has shown that maximum NA activity towards 6'SiaLac does not exceed the activity towards 3'SiaLac, whereas in the work of Baum & Paulson [46], this activity was much higher for the same viruses. It is also worth mentioning that studies of the influenza virus with the simultaneous use of high- and low-molecular weight substrates of a defined structure have yet to be conducted.

Despite the limited amount of data published to date, it is already possible to discuss some features. Firstly, the NA substrate specificity of human isolates differs from that of avian isolates. Secondly, the oligosaccharide specificity of the NA of viruses which circulate in different hosts (birds, pigs, humans) notably differs, at least for the characteristic "ratio of the hydrolysis velocity of 2-3 oligosaccharides towards 2-6 oligosaccharides." Thirdly, the substrate specificity of influenza virus neuraminidases propagated on different cell lines may be different.

Data on NA functioning would be incomplete without taking into account another surface protein of the influenza virus, haemagglutinin. There is only a limited number of publications describing the simultaneous study of HA and NA substrate specificity, and there is virtually no research where the dependence of HA and NA oligosaccharide specificity on one hand and virus infectivity on another hand have been studied. The state-of-the-art analytical procedures for NA introduced in the current review are now up to par with the more advanced analytical methods of HA analysis, which always developed faster; therefore, it is quite easy to predict that one of the main trends in influenza virus studies in the future would be joint studies of HA and NA specificity.
